# Complete Paralysis—Sensation and Motion—Due to Malarial Toxaemia—Report of a Case—Recovery*Read before the Brazos Valley Medical Association, at Marlin, Texas, May 10, 1899.

**Published:** 1899-06

**Authors:** F. R. Collard

**Affiliations:** Wheelock, Texas


For Texas Medical Journal, the Official Organ of the Brazos Valley Medical Association.

### *Complete Paralysis—Sensation and Motion—Due to Malarial Toxaemia—Report of a Case—Recovery.

BY F. R. COLLARD, M. D., WHEELOCK, TEXAS.

While bacteriologists may, and no doubt have, discovered the baccillus or spore on which malarial diseases depend, yet they have not, to my satisfaction, accounted for the varied clinical manifestations of this particular poison, which every practitioner in this climate has observed. They have not told us how this peculiar poison will produce quotidian fever in this instance, tertian in that and quartan in the other. Why we have coma in one patient, delirum in another, or violent vomiting and purging in another; why or how we have the hemorrhagic variety, etc.

It is in direct violence to common sense to assert that one poison, and one alone, will produce all this clinical history. If a toxic dose of strychnia is administered we will expect, confidently expect, convulsions, or ten grains of morphia, coma; or if inoculated with virus of smallpox we expect smallpox; gonococci, gonorrhœa, and so on, ad infinitum.

Now, how is it that this particular poison should present so many pathological conditions? Pathologists tell us it poisons the blood. So we might say of a great many other poisons. What are we going to do with diphtheritic poison, yellow fever poison and a host of others which poison the blood? It may be asked, if it does not poison the blood, what does it poison? i. e., where does it spend its force in the onset? The reply to this question is another story, as Kipling would say, and would require another paper.

I have seen people laboring under the effects of malarial poison for months and months. I have seen others die under its effects in less than twelve hours. I have seen cases of malarial fever in profound coma. I have seen cases who, to all intents and purposes, had cerebro-spinal meningitis. I have seen cases who had an active wild delirium, in full possession of all their muscular power. To such a degree did the delirium extend that the nurses were in danger of personal violence—one case in particular.

G. H.; attacked August 4, with chills and fever. August 5, up

*Read before the Brazos Valley Medical Association, at Marlin, Texas, May 10, 1899.

and dressed, ate breakfast; another light chill about noon; fever followed; semi-comatose in evening. Would answer when spoken to; would get up and sit in a chair, but would pay no attention to house flies, two or three of which were in the corners of his eyes and had their bills out over the sclerotic coat nearly to the cornea. Objected to being prescribed for by a physician; said he would take some pills and quinine that night. I prescribed for him, however, and left the medicine with his brother; he would not take it. Next morning he was up again, but about noon another chill, followed by the usual fever, accompanied by a wild, active delirium. It would take four men to hold him. Would fancy that his nurses were his enemies. Suddenly, as quick as a cat, he jumped from the bed, and in an instant had a shotgun in his hands and the devil in his eye. Fortunately he was overpowered by his attendants. By main force we gagged him and forced him to swallow quinine and calomel—quinine by enema. In forty-eight hours the fever abated and with it the delirium. He was thoroughly cinchonized and made a quick recovery.

But to the case in point—I am diverging from the text. The case of complete paralysis of sensation and motion to all parts below the neck.

W. W. P.; aged 40 years, who had formerly been one of my patrons, but had moved beyond the bounds of my practice, came quite a distance to consult me in reference to himself, with this history: Had had an attack of what he called billious fever in June, one or two attacks in July, same in August. Had recently been taking a tonic—Brown's Iron Bitters—a proprietary medicine—and was under the impression that he was poisoned by the bitters. Said after he had taken a few doses that he had a curious feeling, which he described as a numbness in his leg and foot and in arm and hand of same side, and all on one side of his face, even to his tongue, and that he did not have good use of himself. My diagnosis was partial paralysis due to a broken down condition of the whole system, the result of these various attacks of malarial fever. I prescribed a general tonic of quinine, iron by hydrogen, extract gentian, and strychnine in pill form. This was August 21. August 24, three days later, I was called hurriedly to his bedside. I found him with a temperature of 104, loaded tongue and bound bowels. Had another attack of fever and, while he had full possession of his mental faculties, was completely paralyzed—sensation and motion on both sides from his neck down gone. His fever developed up into a well marked case of malarial fever of remittent type. I remained with him until the 26th. His bowels acted without warning, his urine was retained and I had to resort to catheter. He had fever until September 2, but remained paralyzed; bowels acted without warning, bladder had to be emptied on all occasions with catheter. On the 3rd he was convalescent as far as the fever was concerned; appetite returned. He warned attendants when his bowels would act, but the bladder would still have to be emptied by catheter. He remained in this condition until the 6th. On the 7th he emptied his bladder unaided. I then gave him the tonic which I had prescribed in the onset, and supplemented it with tincture nux vomica until I made the muscles on his face twitch. After the 7th his bowels and bladder were under the control of his will, but the paralysis of all the extremities remained. I kept up the general tonic and brought to my aid electricity (interrupted current). Appetite good, color returning, sleep natural. On the 15th he realized that he could move the great toe on right foot; sensation began to return. He had the nurse to elevate the foot so as he could be able to see it, and was amusing himself when I entered the room by moving it back and forth. Next day he had power over the ankle joint. Three days later was sitting up in a chair. Kept steadily improving and made a complete recovery, and following season made a full hand in the field. This was in 1891. The man is still living and has had no succeeding attacks of paralysis.

Now, the question is, was the paralysis due to malarial poisoning or merely a complication, and due to something else associated with the malarial poisoning? If due to some organic lesion the paralysis would have been permanent, or at least the patient would have had recurring attacks before this time. My experience with paralysis is that one pronounced attack is the beginning of the end after the man passes forty years of age. In this instance my opinion is that it was due to the malarial poison spending its force on the spinal cord. Just what pathological condition existed—whether anæmia or congestion—I am at loss to decide. If it spends its force at one time on the colon and rectum producing malarial dysentery, at another time on the conjunctiva producing malarial conjunctivitis, or at another time on the stomach and bowels, producing vomiting and purging, or at another on the kidney, producing black jaundice, and yet another on the brain, producing coma, why not in some occult manner produce general paralysis?

